# Two-way FDI and corporate total factor productivity--moderating role of environmental information disclosure quality

**DOI:** 10.1371/journal.pone.0342457

**Published:** 2026-03-25

**Authors:** Yu Duan, Yutong Ruan

**Affiliations:** 1 China Center for Special Economic Zone Research, Shenzhen University, Shenzhen, Guangdong, China; 2 Business School, The University of Hong Kong, Hong Kong SAR, China; Shanghai Ocean University, JAPAN

## Abstract

This paper takes A-share Chinese listed companies from 2011 to 2021 as research samples and discusses the effects and potential moderating mechanisms of two-way FDI on corporate total factor productivity. Firstly, It is empirically found that the impact of corporate inward foreign direct investment (IFDI) on its own total factor productivity (TFP) is higher than that brought by outward foreign direct investment (OFDI). After a series of robustness tests, the baseline results are still firm and valid. Secondly, moderating analyses demonstrate that both corporate environmental information disclosure quality and technological innovations exert significant moderating effects on the relationship between two-way FDI and corporate total factor productivity. Thirdly, this paper finds that the effects of two-way FDI faced by listed companies in the central and western regions, non-heavily polluting companies and state-owned listed companies are more significant. Finally, this paper clarifies that policies should be formulated according to local conditions to promote fair competition among enterprises efficiently while reducing the investment and financing thresholds for small enterprises, so as to improve the international competitiveness of Chinese enterprises in various fields as soon as possible.

## 1. Introduction

Since the implementation of China’s opening-up policy at the end of the 1970s, the domestic economy has achieved rapid growth and a leap forward. With the advent of a new stage of development, China’s level of opening up has been further enhanced. This has enabled foreign investors to enter the Chinese market through direct investment at an increasingly faster pace, while domestic investors have also been able to expand their investments and operations overseas through platforms established by the government. This trend has gradually become an integral part of China’s economy [1]. The report of the 20th National Congress of the Communist Party of China emphasized the need to promote high-level opening up, accelerate the construction of a strong trading nation and actively maintain a diversified and stable international economic structure and trade relations. In the new era, some scholars argue that China’s current international economic strategy comprises three dimensions: First, attracting strategic foreign direct investment to adjust China’s economic structure through IFDI, known as the “bringing in” strategy. Second, engaging in outward direct investment (OFDI) to enhance the value, influence and reputation of Chinese enterprises through OFDI, known as the “going out” strategy. Third, optimizing the allocation of the “bringing in” and “going out” strategies to drive comprehensive innovation and economic structural adjustment in China, achieving a virtuous cycle and dynamic optimization between IFDI and OFDI and promoting the transformation and upgrading of China’s economic growth drivers from traditional factor-driven to green innovation-driven [2].

**Fig 1:**
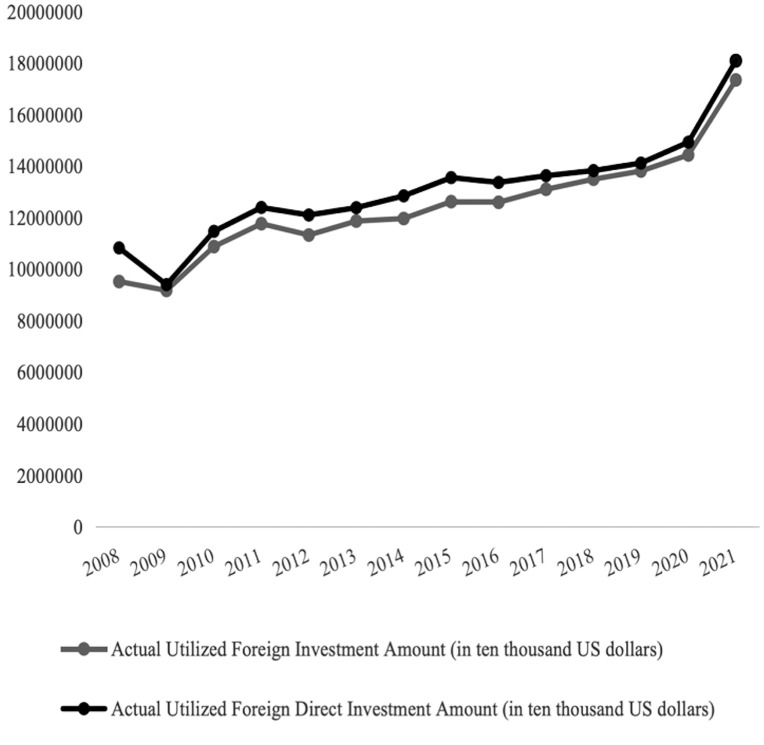
The actual utilization of Foreign Investment in China from 2008 to 2021. Source: The National Bureau of Statistics Website.

**Fig 2:**
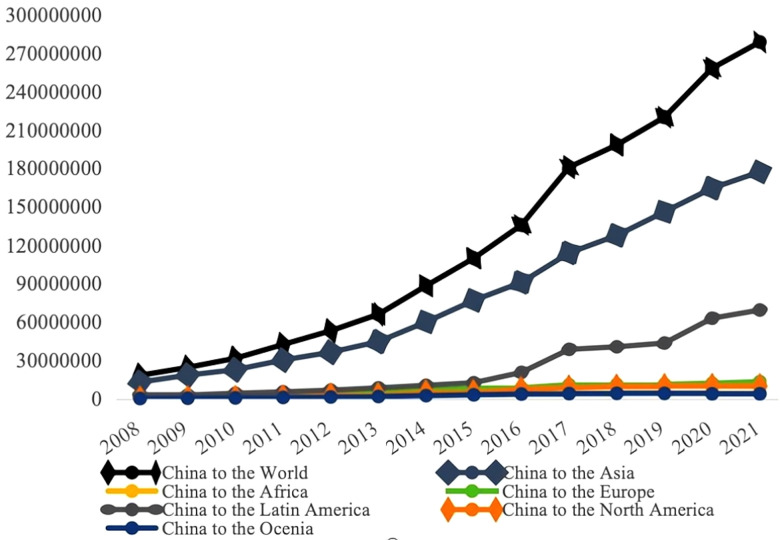
China’s stock of OFDI trend from 2008-2021. Source: The National Bureau of Statistics Website.

Furthermore, some scholars believe that the coordinated development of two-way FDI, characterized by its technological spillover effects and the improvement of capital and labor misallocation, can effectively promote the steady improvement of total factor productivity [3–4]. Figs 1 and 2 respectively illustrate the dynamic trends of China’s two-way FDI investment activities from 2008 to 2021. China’s total outward investment is significantly higher than the amount of actual foreign direct investment utilized. Fig 1 shows that, except for a few years when the global financial crisis had a slight impact on the level of actual foreign capital received, both the actual foreign capital utilized and the actual FDI utilized in China have shown an upward trend. Fig 2 displays the specific situation of China’s active outward investment after the financial crisis. By the end of 2021, China’s stock of direct investment in the world had reached 3 trillion US dollars. In particular, after the 18th National Congress, China further expanded its outward investment and level of openness, resulting in a significant increase in direct investment in Asia and Europe, which accounted for a large proportion of China’s total outward investment.

On November 4 of 2022, President Xi profoundly mentioned at the opening ceremony of the 5th China International Import Expo that openness is an important driving force for the progress of human civilization and a necessary path for the world’s prosperity and development. Less than a year later, on October 18, 2023, President Xi, in his keynote speech at the opening ceremony of the 3rd Belt-and-Road Forum for International Cooperation, stated that China will comprehensively eliminate foreign investment access restrictions in the manufacturing sector [5]. This indicates that, on the basis of the basic opening up of the national manufacturing industry and the complete clearance of the negative list for manufacturing in free trade pilot zones, China has taken another significant step in its opening-up process.

Economic growth is however, a double-edged sword [6]. The contradiction between the rigid constraints of resources and the severe ecological and environmental issues on its reverse side is becoming increasingly acute. At present, China is facing the difficult situation of sluggish economic growth and rising carbon emissions. Therefore, achieving green and low-carbon development has become an intrinsic requirement for high-quality economic development. According to official statistics, China’s per capita carbon emissions are relatively low [7]. The latest data from the World Bank show that in 2021, China’s per capita CO_2_ emissions were 8.4 tons, which is only 72% of the average level of developed countries [8]. However, affected by factors such as the continuous increase in population size and the upward trend in economic development, China’s total carbon emissions first surpassed those of the United States in 2006, making it the world’s largest carbon-emitting country. In recent years, government leaders clearly stated at the 75th session of the United Nations General Assembly the dual goals of “carbon peak” by 2030 and “carbon neutrality” by 2060 [9–10].

At present, China’s internal environmental governance has entered a new stage. Following the implementation of market-incentive and command-and-control environmental regulation policies by the government, environmental information disclosure has become another crucial way to effectively reduce the information asymmetry risk between enterprises, society and the public [11]. In developed countries, environmental information disclosure policies have long been an effective supplement or alternative to direct government environmental control and market instruments [12–14]. China’s environmental information disclosure policy began to be implemented at the end of the 20th century and has gradually been implemented and developed. Listed companies, as active market entities, play an important role in economic growth and the optimization of economic structure. The improvement of their total factor productivity is of paramount importance to achieve efficient, high-quality and sustainable economic development [15]. Based on the view, how to effectively balance the goals of economic growth and achieve green and low-carbon development and meet or even exceed the “carbon peak and carbon neutrality” targets on schedule, is a major test for the long-term development of Chinese enterprises [16–17].

With time going by, does two-way FDI activities significantly promote corporate TFP? And if yes, what are the specific channels of two-way FDI to impact corporate TFP and how to testify the robustness of these ideas? To answer these questions, the paper takes A-share listed companies from 2011 to 2021 as research samples to empirically investigate the impact of firms’ two-way FDI activities on TFP. It also explores the moderating effect of corporate environmental information disclosure between the two and further examines the heterogeneity of the impact. From the perspective of micro enterprises, this study offers some policy recommendations for the government to further promote opening-up and environmental information disclosure policies. It enriches the research on the impact of international trade on corporate production and operation performance and takes into account the role of environmental factors in this process. Firstly, our study aims to untangle the relationships among two-way FDI, TFP, environmental information disclosure and technological innovations and construct a more precise analytical framework that encompasses all core variables. Secondly, by employing the firm-specific level data set, we wish to analyze the specific impact and potential mechanisms of two-way FDI on TFP from an perceptive and subtle firm-level perspective. Lastly, we would like to provide more empirical evidence for the rigorous measurement standard for TFP and figure out whether different measurement methods will ultimately affect the robustness of empirical conclusions. Moreover, research on the construction of corporate environmental disclosure quality systems is also underdeveloped and will be further discussed in the study.

## 2. Literature review

### 2.1. The impact of FDI on TFP

For a long time, the relationship between international trade, environmental pollution and total factor productivity has been a contentious issue in academic circles. Some scholar established a static model of North-South trade to study the relationship between per capita income, environmental pollution and international trade [18]. Since then, the academic community has primarily focused on the impact of capital factor flows on TFP from the perspectives of IFDI and OFDI, either unilaterally or bilaterally. These studies can be categorized into the following three types.

The impact of IFDI on TFP. This discussion mainly originates from debates on hypotheses such as the “Pollution Haven” and “Pollution Refuge”. Developed countries transfer high-pollution industries to host countries through FDI, leading to environmental degradation and pollution [4, 9]. Subsequently, Some economist incorporated IFDI and environmental TFP into a unified analytical framework to study the relationship between IFDI and green TFP [19]. Other researchers used provincial panel data and a mediation model to discuss the regional heterogeneity of the impact of IFDI on TFP through technological innovation as a mediating factor [20]. Scholars employed a spatial Durbin model to investigate the spatial spillover effects of IFDI on green TFP [21]. Some study further utilized macro-level country data to examine the spillover effects of foreign direct investment on the TFP of countries along the Belt and Road Initiative [22].

The second category of studies that discuss the relationship between OFDI and GTFP has reached inconsistent conclusions, which can be summarized into the following three types. The first theory posits that OFDI has a positive impact on GTFP [3, 23]. The second perspective, however, argues that OFDI does not bring about positive effects [24]. The third viewpoint considers both the IFDI and OFDI and their impact on corporate TFP. This view suggests that the reason for the inconsistent conclusions in the previous studies might be due to the failure to consider whether the economic policy environment in which the parent company is situated enables it to obtain reverse technology spillovers from OFDI [25].

### 2.2. The impact of environmental information disclosure quality on firms’ TFP growth

The social demand is the essence of listed firms’ disclosure of their environmental accounting information. At present, the status of environmental information disclosure by listed companies in China cannot effectively meet the objective needs of various stakeholders for economic decision-making. The theoretical basis and motivations for corporate environmental information disclosure are widely discussed in the literature. The main theories include the sustainable development theory, the social responsibility theory and the stewardship theory [26], the signaling theory, the stakeholder theory, the decision-usefulness theory and the information rent theory [27]; the external pressure, moral constraint and social image theories [28]; and the legitimacy or propriety of business operations theory [29].

Meanwhile, the role of FDI inflows in the short-term economic benefits and long-term development of firms is becoming increasingly significant. The inflow and distribution of FDI are important factors in balancing regional economic development structures and making international investment layouts. They are also of great significance for domestic investment, financial deepening and the cultivation of market potential [30]. However, researches about the role of information, especially environmental information disclosure on the impact of two-way FDI on firm’ TFP are relatively scarce, although the existing researches are of high quality. Studies on the impact of environmental information disclosure on total factor productivity is even less frequently mentioned. Based on the mediating effect of technological innovation, some scholar discussed the impact of listed firms’ environmental information disclosure on their TFP [31]. Another interesting research used various spatial econometric models to analyze the dynamic impact of China’s environmental information disclosure network on green TFP [32].

### 2.3. The impact of technological innovation on firms’ TFP growth

Although many researchers address the importance of technological innovations on firm’s short-term and long-term development [33–35], while fewer studies have emphasized on the significance of technological innovations to promote the effect of capital flow on corporate TFP. Venture capital could significantly influence firm innovation performance and further its total factor productivity [36]. Some economist checked the relationship between firm technological and productivity in five Central American countries, and concluded that firm TFP is positively influenced by the credit to firms and firm innovation ability [37]. Some study also examined the role of co-innovation, which is characterized by human capital and training and new forms of work collaboration, on the development of firm’s productivity, finding that co-innovation indirectly elevated firms’ productivity especially in small firms [38]. Another research clarified the effect of listing status on firms’ innovation ability and TFP growth in Chinese industries. The results demonstrated that with initial public offering, firms bear ascending on innovation input while descending on innovation output, which further dampened firms’ TFP development.

Existing studies predominantly employ provincial panel data to investigate the static or bidirectional dynamic effects of foreign direct investment on TFP or green TFP at the provincial or major regional levels. Additionally, numerous studies have examined the impact of IFDI on TFP or GTFP across different industries. Some scholars have also approached the key variables from a more macroscopic national perspective. To sum up, the existing literature has several aspects that could be improved.

Firstly, the existing literature focuses on the impact relationships between any two or three of these variables, which are environmental information disclosure, technological innovation, TFP and FDI, yet fails to construct a more precise analytical framework that encompasses all core variables. As a result, the complex interactive mechanisms among these crucial variables remain unclear. Secondly, the majority of existing studies analyze the impact of FDI on TFP from a macroscopic national or provincial level or a meso-level industry perspective. On the contrary, analyses of key economic variables at the micro-level firm-specific level are relatively scarce. Lastly, the academic community has not yet established a unified measurement standard for TFP. There is still considerable debate over whether different measurement methods will ultimately affect the robustness of empirical conclusions. Moreover, research on the construction of corporate environmental disclosure quality systems is also underdeveloped and requires further discussion.

## 3. Theoretical analysis

### 3.1. Inward foreign direct investment and TFP of firms

With the continuous deepening of China’s opening-up process, the long-term development effect of foreign investment on the national economy and its promoting effect on further reform have become increasingly evident. Therefore, it is particularly crucial to conduct an in-depth investigation into the role of FDI in the long-term development of enterprises.

From a theoretical perspective, on the one hand, the continuous inflow of foreign capital can effectively disperse the risks induced by the uncertainty of corporate innovation to a certain extent and further encourage enterprises to actively engage in research and development (R&D) and innovation activities. This, in turn, can enhance the competitiveness of enterprises in terms of technological innovation [39] and human capital [40]. On the other hand, the signaling function inherent in FDI can enable enterprises to obtain more financing incentives [41], especially in regions with highly developed financial systems. FDI can not only promote economic growth by improving the total factor productivity of enterprises and even the entire region but also achieve economic takeoff through the accumulation of factors.

In terms of practical application, firstly, FDI creates market competition that stimulates domestic firms to continuously improve their management efficiency and factor utilization rates and further increase their R&D efforts, thus enhancing their TFP. However, at the same time, the inflow of IFDI also exerts a certain crowding-out effect on domestic enterprises. Secondly, the new knowledge or technology brought by IFDI can be imitated and referenced by domestic firms, thus promoting the improvement of production technology, marketing models and management methods and, consequently, increasing productivity. Thirdly, foreign firms provide employment training for local employees and internalize human capital within individual workers. The mobility of employees between different firms leads to the outflow of human capital. Moreover, foreign and domestic firms in the same industrial chain, due to cooperation needs, jointly possess unified technology and production standards for joint development. This also helps to enhance the TFP of domestic firms. However, FDI may also bring some externality, such as environmental pollution, social issues, or quality decline. Based on the above analysis, this paper proposes the following hypothesis H1:

H1: Foreign direct investment in enterprises has a certain promoting effect on the total factor productivity of enterprises.

### 3.2. Outward foreign direct investment and TFP of firms

From the perspective of corporate microeconomics, enterprises that engage in OFDI obtain reverse technology spillovers through four means: emulating and referencing the production and R&D processes of foreign firms, embedding themselves in the R&D agglomeration networks of foreign firms through forward and backward linkages, introducing high-end, versatile human capital from abroad and leveraging platforms for the agglomeration of foreign technology and industries [22]. On a broader macroeconomic level, OFDI by domestic firms into developed countries is primarily conducted through cross-border mergers and acquisitions or R&D organizations to acquire high-tech and subsequently repatriate it. OFDI to other developing countries, however, mainly involves driving net exports of upstream products and local production to achieve economies of scale and increase R&D costs [23]. Moreover, enterprises inevitably face intense competition from foreign firms when engaging in OFDI. This, in turn, stimulates the intent of domestic investing firms to actively safeguard their competitive edge in overseas markets. Consequently, they adopt a technology-biased sustainable development model. Through reverse technology transfer channels, high-end technologies are fed back to the home country, effectively prompting domestic firms to engage in energy-saving, emission-reducing and green technological innovation. Meanwhile, as enterprises transfer some excess capacity during the OFDI process, the economic development factors in the home country become more cohesive. This further raises the entry barriers for IFDI in the domestic market, optimizes economic development outcomes and facilitates the mobility of high-tech personnel as well as the spillover effects of technology. Based on the above analysis, this paper proposes the following hypothesis H2:

H2: Outward foreign direct investment by enterprises has a positive promoting effect on the total factor productivity of enterprises.

### 3.3. Two-way FDI and TFP of firms

At present, the majority of scholars concur that the complementary effect of two-way FDI can significantly enhance total factor productivity. When a firm simultaneously engages in IFDI and OFDI activities, although the inward direct investment may have a substantial negative impact on the local environmental quality, it is generally believed that the continuous expansion of capital stock and production scale exerts a significant driving force on the local economy. Moreover, the two-way FDI activities undertaken by enterprises manifest as the dual flow of factors such as capital, technology, talent and management experience. The positive spillover effects of mutual learning are evident. Additionally, as the performance and economic strength of domestic enterprises improve, the environmental entry barriers for IFDI are raised during the expansion of OFDI activities, thus effectively curbing the negative impacts of IFDI. In conclusion, the two-way FDI investment activities of enterprises can jointly influence the improvement and change of total factor productivity through three aspects: scale effect, technology effect and structural effect. Based on the above analysis, this paper proposes the following hypothesis H3:

H3: The synergy of two-way FDI has a positive effect on the improvement of total factor productivity of enterprises.

### 3.4. Mechanism analysis of the two-way FDI on corporate TFP

For listed companies, engaging in environmental information disclosure is a crucial metric for stakeholders to effectively assess the attitude and capability of firms in fulfilling their social responsibilities. Given that firms often cause severe environmental pollution during the production process, it is imperative to focus not only on the improvement of economic benefits but also on potential pollution and environmental technological innovation in daily operations. On the one hand, when disclosing information, firms tend to highlight positive green practices, such as innovations in green production technology. However, this may crowd out other non-green investments, leading to increased production costs and potentially lower profit margins. On the other hand, firms with high-quality environmental disclosures are generally perceived by potential investors as more capable and socially responsible, thus receiving a positive environmental risk premium that effectively reduces their financing costs. Therefore, the quality of corporate environmental information disclosure has multiple and complex effects on the relationship between two-way FDI and total factor productivity.

When it comes to the firms’ technological innovations, on one hand, firms receive inward investment from technological advancement, which further helps to moderate the impact process of FDI on corporate TFP. On the other hand, firms’ innovation ability could facilitate reverse knowledge spillovers and transfer knowledge and important information back, thus effectively promote long-term growth and development. Moreover, technological innovations could essentially mitigate the potential operation cost and risk especially for firms living in an fast-changing and competitive society.

Based on the above analysis, this paper proposes Hypothesis H4.

H4: The quality of corporate environmental information disclosure and technological innovation ability positively moderate the relationship between two-way FDI and corporate total factor productivity.

## 4. Data and materials

### 4.1. Data

This study employs A-share listed companies in China from 2011 to 2021 as the research sample. Firm-level and industry-level data are sourced from the CSMAR database and the Statistical Bulletin of China’s Outward Foreign Direct Investment published annually by the Ministry of Commerce. The sample excludes: (1) firms labeled with ‘ST’ or ‘*ST’ (indicating abnormal financial status); (2) firms with negative net assets, a debt-to-asset ratio exceeding 1, or negative net profits; and (3) firms with severe missing data. To mitigate the potential bias caused by outliers, continuous variables are winsorized at the 1% level.

### 4.2. Variable measurement

#### 4.2.1. Explained variable: Total factor productivity.

**Fig 3:**
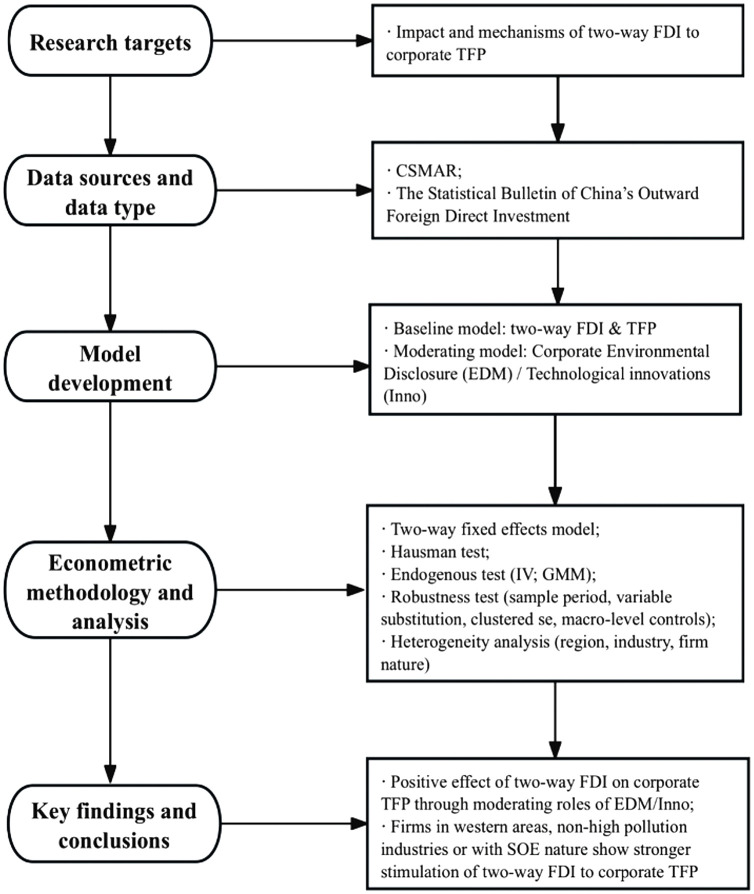
Conceptual flowchart of the empirical research.

Total factor productivity, a comprehensive metric of firm efficiency, was initially proposed [42]. It is measured as the “Solow residual”, representing the portion of output not explained by inputs of labor and capital. While straightforward, this method suffers from endogeneity and selection bias issues. The conceptual flowchart of the research is presented as follows (Fig 3).

To address limitations in fixed-effects approaches, economists introduced a consistent semi-parametric estimator, which gained widespread adoption and was called OP method [43]. This method assumes that firms adjust investment in response to productivity changes. However, the OP method requires a critical assumption: a monotonic relationship between the proxy variable (investment) and output, leading to its unavailable to firms with zero investment. Other scholars proposed an alternative approach using intermediate inputs as proxies for TFP estimation, which was called LP method [44]. The LP method is more flexible because intermediate inputs such as raw materials are commonly used by all firms, even those with zero investment. Therefore, this study employs the LP method to estimate TFP (*TFP_LP*) as the dependent variable in baseline regressions, while TFP estimated via the OP method (*TFP_OP*) serves as a replacement variable for robustness checks. The LP estimation model is specified as follows:


$$\text{In}Y_{i,t}=\;a_{\text{0}}\;+\;a_{\text{1}}\text{ In }L_{i,t}\;+\;a_{\text{2}}\text{ In }K_{i,t}\;+\;a_{\text{3}}\text{ In }{Mid}_{i,t}\;+\sum year\;+\;\sum Ind\;+\epsilon_{i.t}\;$$ (1)

In the model, *i* denotes listed firms, *t* represents time, *LnY* is the natural logarithm of a firm’s core business revenue, *LnL* is the logarithm of the year-end number of employees and *LnK* is the logarithm of the net fixed assets of the listed firm. *LnMid* serves as the proxy variable for intermediate inputs, calculated as the sum of six accounting items from financial statements (Intermediate Inputs = Cost of Revenue+Administrative Expenses+Financial Expenses-Depreciation of Fixed Assets-Amortization of Intangible Assets- Cash Paid to and for Employees). Additionally, *Year* and *Ind* represent the year and industry fixed effects controlled in the model, respectively, while *ε* denotes the random error term.

#### 4.2.2. Core explanatory variable: Two-way FDI (IFDI & OFDI).

The core explanatory variable, two-way FDI, encompasses both IFDI and OFDI. Existing literature typically measures foreign capital utilization using dummy variables, such as whether a firm is foreign-owned [45], the proportion of foreign shares in equity structure, or the attributes of the ultimate controller. However, while dummy variables effectively capture firm ownership, they fail to reflect firms’ inner variations in FDI capital flows. To address this limitation, this study adopts the previous methodology [46], measuring IFDI as the natural logarithm of the sum of 1 and the product of the proportion of foreign ownership among the top ten shareholders multiplied by the firm’s registered capital. Similarly, OFDI is measured as the logarithm of the total outward investment amount. All continuous variables are smoothed by taking the natural logarithm.

**Fig 4:**
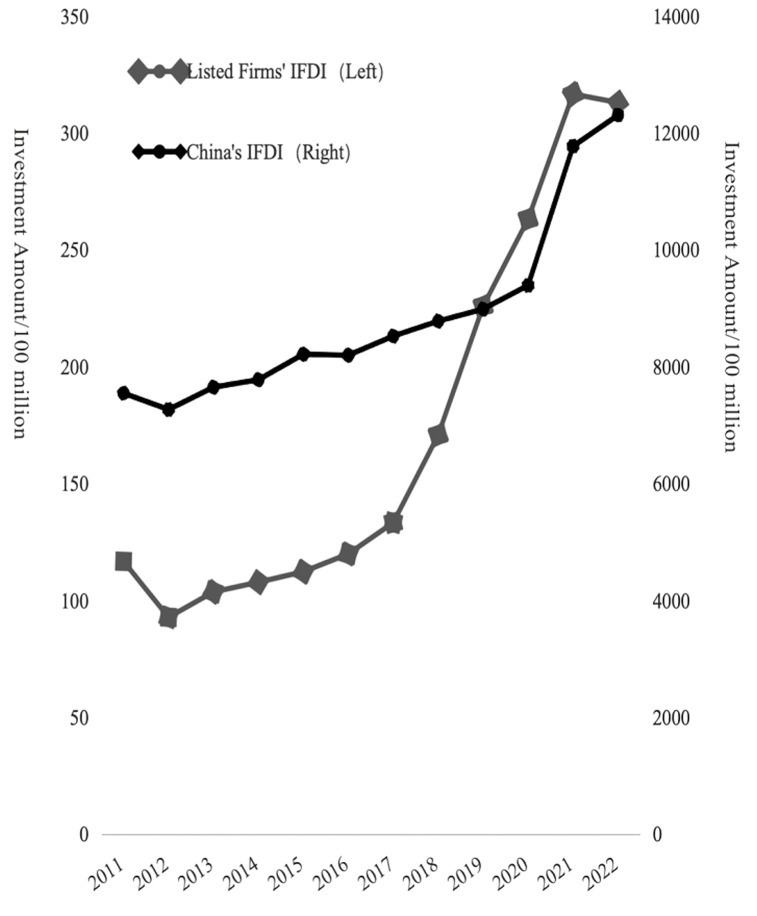
China’s listed firms’ IFDI trend from 2011-2022. Source: The National Bureau of Statistics Website.

**Fig 5:**
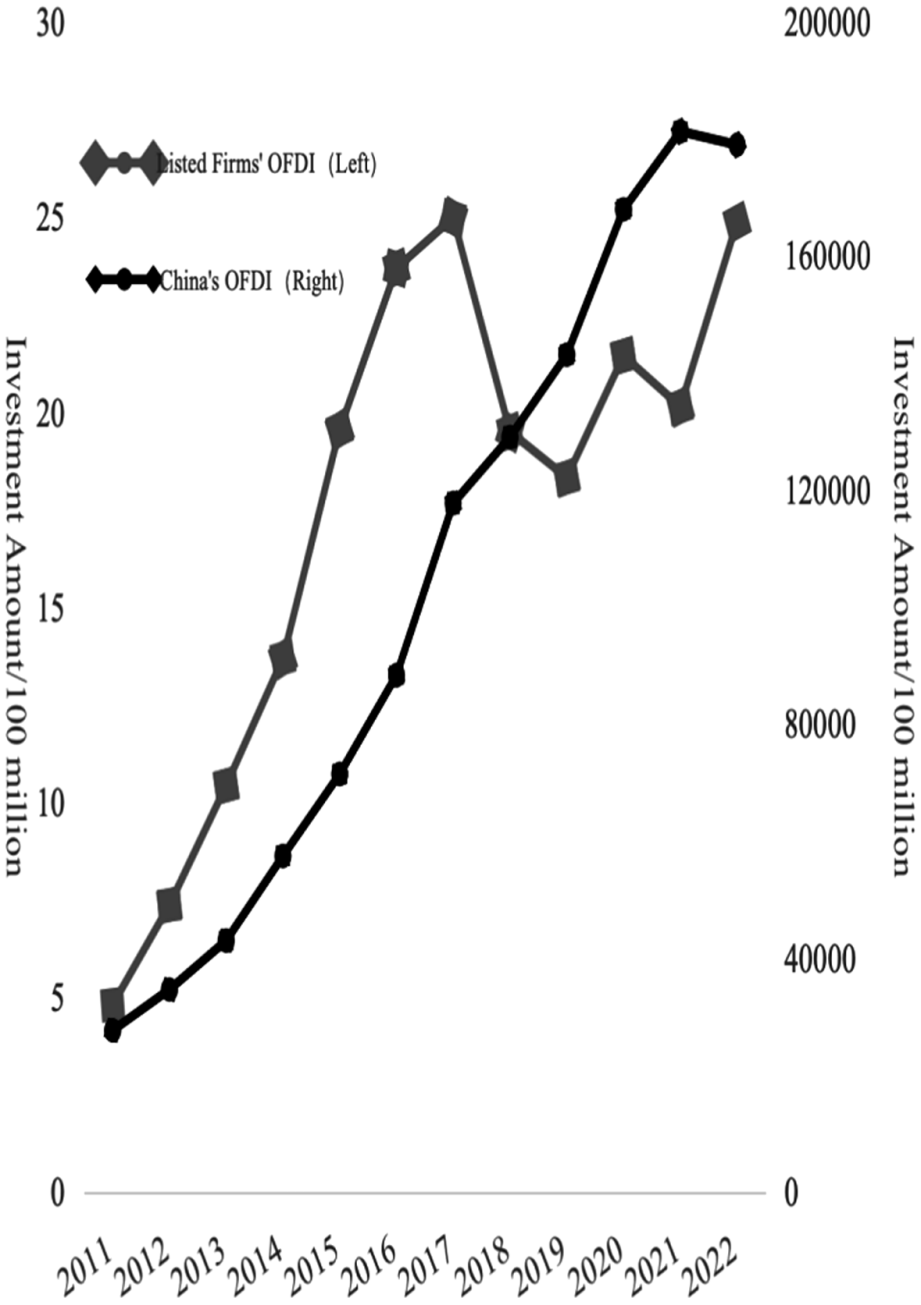
China’s listed firms’ OFDI trend from 2011-2022. Source: The National Bureau of Statistics Website.

The final data set comprises 24438 firm-year observations from 3664 listed firms spanning 2011–2021. To assess the correctness of the two-way FDI sample, a preliminary comparison was conducted between the trends of FDI flows in the sample and China’s aggregate FDI data. As illustrated in Figs 4 and 5, the two-way FDI trends of listed firms closely align with the national-level trends, suggesting that the constructed sample reliably reflects China’s two-way FDI dynamics. This further implies that the listed firm sample serves as a valid proxy for analyzing the broader evolution of China’s FDI landscape.

#### 4.2.3. Moderating variable: Quality of corporate environmental disclosure (EDM) and technological innovations (Inno).

This study employs the Corporate Environmental Disclosure Index (*EDM*) as a moderating variable to quantify the level of corporate environmental disclosure. Based on data availability and operational feasibility and following the methodologies [47], this paper adopts the environmental disclosure framework from the CSMAR database. For the first three tables, including the Environmental Management Disclosure Table, the Environmental Regulation and Certification Disclosure Table and the Environmental Information Disclosure Medium Table, a score of 2 is assigned if substantive content is disclosed, otherwise 0. To be more specific, the Environmental Liability Disclosure Table includes six items: wastewater, COD, SO_2_, soot and dust emissions and industrial solid waste generation. The Environmental Performance and Governance Disclosure Table includes six items: waste gas and wastewater treatment, dust and soot control, solid waste utilization and disposal, noise, light pollution and radiation management and clean production implementation. For the Environmental Liability Disclosure Table and Environmental Performance and Governance Disclosure Table, which comprise 12 indicators in total, each indicator is scored as follows: 2 points for both qualitative and quantitative descriptions, 1 point for qualitative descriptions only and 0 points otherwise. The maximum total score is 30 and the minimum is 0. The final moderating variable *EDM*, is calculated as the natural logarithm of the sum of the scores across all 15 indicators plus 1. The exact measurement of EDM index could be seen in Table 1. When it comes to corporate technological innovations, referring to existing literature [48–49], this paper employs the number of innovation patent applications filed annually by firms as the measurement indicator of technological innovation output.

**Table 1:** Measurement of corporate environmental disclosure (EDM).

Goal	Framework	Indicator	Standard
Corporate Environmental Disclosure Index	Environmental Management Disclosure Table	/	Two scores if substantive content is disclosed, otherwise 0
	Environmental Regulation and Certification Disclosure Table	/	
	Environmental Information Disclosure Medium Table	/	
	Environmental Liability Disclosure Table	Wastewater	Two scores for both qualitative and quantitative descriptions, one score for qualitative descriptions only and 0, otherwise.
		COD	
		SO_2_	
		Soot	
		Dust emissions	
		Industrial solid waste generation	
	Environmental Performance and Governance Disclosure Table	Wastewater treatment	
		Waste gas treatment	
		Dust and soot control	
		Solid waste utilization and disposal,	
		Noise, light pollution and radiation management	
		Clean production implementation.	

#### 4.2.4. Control variables.

To effectively control for other firm-level characteristics that may influence *TFP*, the following control variables are included, drawing on the work [36]. Firm Size (*Size*) is measured as the natural logarithm of total assets at year-end. Ownership Concentration (*Center*) is represented by the combined shareholding percentage of the top ten shareholders. Firm Nature (*SOE*) means a dummy variable where state-owned enterprises are assigned 1 and non-SOEs take the value of 0. Debt-to-Asset Ratio (*DA*) is calculated as total liabilities divided by total assets. Capital Liquidity (*Flow*) is defined as current assets-current liabilities divided by total assets. Return on Assets (*Return*) is measured as operating profit divided by total assets. Fixed Asset Ratio (*F_ratio*) is Calculated as net fixed assets divided by total assets. Firm Age (*Age*) is quantified as the current year minus the year of establishment. The definitions and notations of these variables are summarized in Table 2.

**Table 2:** Variable and symbol.

Variable	Symbol	Definition	Source
Independent variable	*TFP*	The natural logarithm of total factor productivity calculated using the LP method and OP method.	Calculate manually
Core independent variable	*IFDI_mi*	The natural logarithm of the foreign shareholding proportion among the top ten shareholders multiplied by the registered capital	CSMAR
	*OFDI_mi*	The natural logarithm of the total outward investment of the enterprise.	CSMAR
Moderating variable	*EDM*	The sum scores from the 15 environmental disclosure evaluation indicators for listed companies.	CSMAR
	*Inno*	The natural logarithm number of patent applications filed annually by firms.	CNRDS
Control variable	*Size*	The natural logarithm of the firm’s total assets at year-end.	CSMAR
	*Center*	The combined shareholding ratio of the top ten shareholders of the company.	CSMAR
	*DA*	The ratio of total liabilities to total assets.	CSMAR
	*Flow*	(Current Assets – Current Liabilities)/ Total Assets.	CSMAR
	*Return*	The ratio of operating profit to total assets.	CSMAR
	*F_ratio*	The ratio of net fixed assets to total assets.	CSMAR
	*Age*	The time since the establishment of the enterprise.	CSMAR
	*SOE*	State-owned enterprises (SOEs) are coded as 1, while non-SOEs are coded as 0.	CSMAR

## 5. Model development and methodology

### 5.1. Model selection

In this section, initially we try to figure out which econometric model can fit well in the study of the production effects of two-way FDI. The core assumption of models with fixed effects is that the invariant individual characteristics is correlated with the independent variables. By using within-group deviations or by including individual fixed effects to partial out the influence of these characteristics, we can obtain a consistent estimate of the regression effects [50]. On the contrary, models with random effects suppose there is no correlation between the invariant individual characteristics and the independent variables. Hausman test is commonly used in empirical studies to distinguish whether the invariant individual effects have correlation with the independent variables of the study [51, 52]. Table 3 displays the results of Hausman tests for both random effects and two-way fixed effects including year-fixed effects and industrial-fixed effects. The p value is 0, which indicates that two-way fixed effects model fits better than the random effects one does.

**Table 3:** Hausman test.

	(b) re	(B) Fe2	(b-B) Difference	Sqrt(diag(V_b -V_B)) Std. Err.
IFDI_mi	.0141973	.0339276	−.0197303	.0007367
OFDI_mi	.0046229	.0194396	−.0148166	.0008657
IFDI_mi*OFDI_mi	−.0004363	.0004039	−.0008402	.0001732
Center	.2242187	.2614085	−.0371898	.0281876
DA	4.750586	9.323444	−4.572858	.1728709
Flow	.8057227	2.415615	−1.609892	.1350735
Return	.1614503	.2761101	−.1146599	.0045987
F_ratio	−1.470046	−.9489339	−.5211117	.0656485
Age	.0248522	.0027599	.0220923	.0006248

Prob > chi2 = 0.0000.

### 5.2. Model setting

Up till now, similar with the results of Hausman tests in our research, plenty of studies have already utilized econometric models with several fixed effects in the international trade and innovation development [3, 52–54]. Thus, we incorporate IFDI and OFDI, as well as their interaction term, into the empirical regression model with two-way fixed effects. We construct the benchmark models to examine the relationship between firms’ levels of IFDI, OFDI, two-way FDI and TFP as shown in Equation (2):


$${\text{TFP}}_{\text{i},\text{t}}=\alpha_{\text{0}}+\alpha_{\text{1}}{\text{IFDI}}_{\text{i},\text{t}}+\alpha_{\text{2}}{\text{OFDI}}_{\text{i},\text{t}}+\alpha_{\text{3}}{\text{IFDI}}_{\text{i},\text{t}}\times {\text{OFDI}}_{\text{i},\text{t}}+\eta {\text{X}}_{\text{i},\text{t}}+\delta_{\text{t}}+\gamma_{\text{i}}+\varepsilon_{\text{i},\text{t}}$$ (2)

In the model, *i* denotes the firm and *t* represents the time period. *X* is a vector of control variables that may influence the total factor productivity of firms. *η* represents the coefficients of the control variables. *δ* and *γ* respectively denote the fixed effects for year and industry and *∊* is the random disturbance term.

The moderating role of the information disclosure quality and technological innovations of listed firms on the relationship between two-way FDI and total factor productivity is also of significant interest. This study sets up Model (3) to investigate the magnitude of this moderating effect. Here, FDI represents the combined effect of IFDI, OFDI and their interaction terms. Based on the continuous dynamic data of A-share listed firms from 2011 to 2022, this study delves into the specific interactive relationships among these variables.


$${\text{TFP}}_{\text{i},\text{t}}=\beta_{\text{0}}+\beta_{\text{1}}{\text{FDI}}_{\text{i},\text{t}}+\beta_{\text{2}}{\text{FDI}}_{\text{i},\text{t}}\times {\text{Z}}_{\text{i},\text{t}}+\eta {\text{X}}_{\text{i},\text{t}}+\delta_{\text{t}}+\gamma_{\text{i}}+\varepsilon_{\text{i},\text{t}}$$ (3)

In this model, *i* denotes the firm and *t* represents the time period. *Z* is the moderating variables including both information disclosure quality and technological innovations of listed firms. *X* is a vector of control variables that may influence the total factor productivity of firms. *η* represents the coefficients of the control variables. *δ* and *γ* respectively denote the fixed effects for year and industry and *∊* is the random disturbance term.

Furthermore, to confirm the validity of the empirical model, we report the results of unit root as the pre-diagnostic tests. Table 4 illustrates that the p-values of Fisher-ADF tests for main variables in the study are all smaller than 0.05. Thus, the panel data we employ in the study are valid and firm.

**Table 4:** Unit root tests.

Fisher-ADF	TFP	IFDI_mi	OFDI_mi	Center	DA	Flow	Return	F_ratio	Age
Lag (1)	0.000	0.000	0.000	0.000	0.000	0.000	0.000	0.000	0.000
Lag (2)	0.000	0.000	0.000	0.000	0.000	0.000	0.000	0.000	0.000

Note: Numbers are p-value.

### 5.3. Descriptive statistics

The descriptive statistics of the main variables are presented in Table 5. The Variance Inflation Factor (VIF) values for all variables are well below 10, indicating the absence of multi-collinearity issues among the variables. As shown in the table, after logarithmic transformation and winsorization, the standard deviation of total factor productivity for listed firms is 0.447, which is relatively small. This suggests that there is not much variation in economic efficiency among A-share listed firms as a whole.

**Table 5:** Descriptive statistics and VIF analysis.

Symbol	Obs.	Mean	SD	Min	Max	VIF
*TFP*	24438	6.830	0.447	5.965	8.094	/
*IFDI_mi*	24438	2.052	3.350	0	9.286	1.14
*OFDI_mi*	24438	2.213	2.399	0	6.176	1.02
*EDM*	24438	8.392	4.702	32	23	1.17
*Inno*	24438	2.102	1.622	0	9.087	1.18
*Center*	24438	1.772	0.120	1.340	1.965	1.11
*DA*	24438	0.951	0.030	0.860	0.995	5.17
*Flow*	24438	0.0311	0.0353	−0.0484	0.134	5.60
*Return*	24438	0.799	0.232	0	0.940	1.09
*F_ratio*	24438	0.914	0.0457	0.740	0.984	1.31
*Age*	24438	19.204	5.985	0	65	1.08

In terms of two-way FDI, the vast majority of firms have engaged in investment activities according to the measurement criteria used in this study. However, a small proportion of firms, due to geographical location or their own developmental characteristics, have zero inward or OFDI, which is not conducive to their long-term efficient development. Echoing this finding, the standard deviation of environmental information disclosure quality for listed firms is 4.702, indicating significant differences in the transparency of various environmental practices adopted by different firms during their development. This may partly explain the moderating mechanism and effect of environmental information disclosure on the relationship between two-way FDI and corporate economic efficiency. The statistical results of other control variables are generally reasonable and are consistent with existing research [35,38,48–49,55], so they will not be reiterated here.

## 6. Empirical studies

### 6.1. Baseline regression

The benchmark regression results are displayed in Table 6. To mitigate measurement error, all models include a set of control variables. To address potential issues of heteroscedasticity and auto-correlation, robust standard errors are employed in all regressions.

**Table 6:** Benchmark Model.

Variable	(1) *TFP_LP*	(2) *IFDI_mi*	(3) *OFDI_mi*
*EDM*		0.0851*** (0.0163)	0.0716*** (0.0122)
*EDM2*		0.0014** (0.0007)	−0.0008 (0.0005)
*IFDI_mi*	0.0339*** (0.0009)		
*OFDI_mi*	0.0194*** (0.0010)		
*IFDI_mi·OFDI_mi*	0.0004* (0.0002)		
Controls	YES	YES	YES
Industrial-fixed effect	YES	YES	YES
Year-fixed effects	YES	YES	YES
R^2^	0.5281	0.1313	0.0862
Obs.	24438	24438	24438

Notes: (1) The numbers in parentheses are robust standard errors; (2) *, ** and *** indicate significance at the 10%, 5% and 1% levels, respectively.

Models (1) reveals that both inward foreign direct investment and outward foreign direct investment exert a positive and significant impact on firms’ TFP at the 1% significance level. The coefficients of the impact of inward IFDI, outward FDI and two-way FDI on total factor productivity are 0.0399, 0.0194 and 0.0004, accordingly. It could be easily seen that the effect of OFDI on TFP is slightly smaller than that of IFDI, suggesting that on average, Chinese listed firms’ inward investments are more conducive to enhancing their productivity than OFDI. This finding implies that IFDI can introduce new technologies and management practices that improve production efficiency. Meanwhile, it reflects that firms’ outward investment or financing activities significantly boost their economic efficiency and development, generating a reverse technology spillover effect, but still in an initial stage.

Models (2) and (3) examine the relationship between IFDI and OFDI and firms’ environmental information disclosure quality. According to the Environmental Kuznets Curve (EKC) theory, when a country is underdeveloped, environmental pollution is relatively mild. However, as per capita income rises, pollution levels increase, worsening environmental degradation. Once the economy reaches a certain threshold or “turning point”, further increases in per capita income lead to a decline in pollution levels and an improvement in environmental quality. Model (3) shows that the relationship between OFDI and environmental information disclosure quality follows an inverted “U” shape. As firms enhance their environmental information disclosure, they possess more confidence to expand the oversea markets and increase OFDI investment. However, after reaching the “turning point” of 44.75, further improvements in disclosure quality result in a decline in OFDI. This suggests that excessive disclosure of key information related to firm development may cause unnecessary concerns for investors. In comparison, Model (2) reveals a “U”-shaped relationship between IFDI and environmental information disclosure quality. And because there is no “turning point” in this curve, the impact of firms’ environmental information disclosure on its IFDI is always positive. This indicates that the more corporate disclosure their environmental information, the more detailed messages could be sent to other firms and investors, leading to less information asymmetry and more investment afterwards.

### 6.2. Endogenous tests

The presence of endogeneity may lead to biased estimation results, which is generally due to omitted variables or reverse causality. To further address the endogeneity caused by omitted variables, we increase the number of control variables. In the subsequent model, we introduce lagged terms of the control variables to mitigate the issue of reverse causality.

Geographical location generally affects the ability of firms and regions to attract foreign investment and engage in outward investment. Firms and areas closer to the coastline tend to have better transportation accessibility. Moreover, the closer a firm is to a port city, the lower its transportation and information transmission costs to a certain extent. Therefore, this paper selects two dummy variables, including whether the registered location of a listed firm is in a port city (*Port*) and whether it is in an eastern coastal province (*East*) to conduct separate model regressions. And we multiply these two dummy variables with corporate’ both inward FDI and outward FDI, making the interaction section be the instrumental variables. The results are shown in Table 7. The endogeneity test results show that the empirical regression is consistent with the benchmark model regression results, further confirming the conclusions of the benchmark regression. Specifically, the coefficients of IFDI and OFDI aligns with the benchmark regression results and all pass the LM test and Wald F test, reducing the endogeneity risk of the model.

**Table 7:** Endogenous Test.

Variable	(1) *IFDI_mi*	(2) *TFP_LP*	(3) *OFDI_mi*	(4) *TFP_LP*	(5) *IFDI_mi*	(6) *TFP_LP*	(7) *OFDI_mi*	(8) *TFP_LP*
*IFDI_mi*		0.0095^**^ (0.0006)		0.0094^***^ (0.0005)		0.0105^***^ (0.0008)		0.0094^***^ (0.0005)
*OFDI_mi*		0.0023^***^ (0.0006)		0.0012^*^ (0.0007)		0.0024^***^ (0.0006)		0.0026^***^ (0.0009)
*East*	0.9531^***^ (0.0049)		0.9857^***^ (0.0048)					
*Post*					0.9280^***^ (0.0083)		0.9957^***^ (0.0086)	
Controls	YES	YES	YES	YES	YES	YES	YES	YES
Anderson canon. corr. LM statistic	13497.30 [0.0000]		14050.68 [0.0000]		7808.221 [0.0000]		8182.35 [0.0000]	
Cragg-Donald Wald F statistic	37648.58		42296.97		12000		13385.96	
Obs	24073	24073	24073	24073	24073	24073	24073	24073

Notes: (1) The numbers in parentheses are robust standard errors; the numbers in square brackets are p-values; (2) *, ** and *** indicate significance at the 10%, 5% and 1% levels, respectively.

Moreover, this paper applies the IV-GMM method to further verify the correctness of the model. Considering that firms’ total factor productivity has a certain degree of time lag, this study lags the dependent variable of total factor productivity and predetermined variables, related endogenous variables and exogenous variables by a certain number of periods before regression. The regression results are displayed in Table 8. It can be derived that Models (1)-(3) verify that the disturbance term has no auto-correlation and all pass the Hansen test, with significance levels basically consistent with the benchmark regression results. To be more specific, the p-values of each model are all between 0.1 and 0.25, demonstrating that the IV variables are not only exogenous but also enough to back up the GMM robustness test. The p-value for AR(1) is less than 0.05, indicating the presence of first-order auto-correlation, necessitating further model adjustments or the use of difference transformations. The p-value for AR(2) is greater than 0.05, indicating the absence of second-order auto-correlation and eliminating the need for further testing of higher-order differences. The GMM regression results are consistent with the benchmark model, easing the endogenous risk of the benchmark regression.

**Table 8:** GMM results.

Variable	(1) *TFP_LP*	(2) *TFP_LP*	(3) *TFP_LP*	(4) *IFDI_mi*	(5) *OFDI_mi*
*L.TFP_LP*	0.137^***^ (0.0365)	0.332^***^ (0.0480)	0.736^***^ (0.0355)		
*EDM*				0.703^***^ (0.194)	−13.307^**^ (2.210)
*EDM2*				−0.0306^***^(0.00869)	0.614^***^ (0.104)
*IFDI_mi*	0.0115^**^ (0.00510)				
*OFDI_mi*		0.0464^***^ (0.0168)			
*IFDI_mi·OFDI_mi*			0.000497^***^ (0.000144)		
*L.IFDI_mi*				0.760^***^ (0.0440)	
*L.OFDI_mi*					−0.195^***^ (0.0814)
Controls	YES	YES	YES	YES	YES
Industrial-fixed effect	YES	YES	YES	YES	YES
Year-fixed effects	YES	YES	YES	YES	YES
AR(1)	−7.02^***^ [0.000]	−8.91^***^ [0.000]	−9.61^***^ [0.001]	−20.07^***^ [0.000]	−5.91^***^ [0.000]
AR(2)	0.04 [0.967]	0.81 [0.415]	−0.93 [0.352]	6.35 [0.000]	−2.39 [0.017]
Hansen test	7.99 [0.157]	13.06 [0.220]	13.92 [0.237]	15.42 [0.118]	19.24 [0.116]
*Obs*	24438	24438	24438	24438	24438

Notes: (1) The numbers in parentheses are robust standard errors; the numbers in square brackets are p-values; (2) *, ** and *** indicate significance at the 10%, 5% and 1% levels, respectively.

### 6.3. Robustness tests

To effectively verify the accuracy of the benchmark regression, this section employs several methods for robustness checks, including changing the sample period, replacing the dependent variable, replacing the core independent variable and employing GMM method. The results are shown in Tables 9–11.

**Table 9:** Robustness Test I.

Variable	(1) *TFP_LP*	(2) *TFP_LP*	(3) *TFP_LP*	(4) *TFP_OP*	(5) *TFP_OP*	(6) *TFP_OP*
*IFDI_mi*	0.0415*** (0.0015)	0.0396*** (0.0013)	0.0310*** (0.0011)	0.0240*** (0.0010)	0.0248*** (0.0009)	0.0195*** (0.0007)
*OFDI_mi*	0.0265*** (0.0015)	0.0220*** (0.0013)	0.0221*** (0.0011)	0.0157*** (0.0012)	0.0136*** (0.0011)	0.0118*** (0.0009)
*IFDI_mi·OFDI_mi*	0.0012*** (0.0004)	0.0011*** (0.0004)	0.0008*** (0.0003)	−0.0011*** (0.0003)	−0.0009*** (0.0003)	−0.0009*** (0.0002)
Controls	NO	NO	YES	NO	NO	YES
Industrial-fixed effect	NO	YES	YES	NO	YES	YES
Year-fixed effects	YES	YES	YES	YES	YES	YES
R^2^	0.1423	0.3253	0.5222	0.0593	0.2797	0.5428
Obs.	18001	18000	17999	24440	24439	24438

Notes: (1) The numbers in parentheses are robust standard errors; (2) *, ** and *** indicate significance at the 10%, 5% and 1% levels, respectively.

**Table 10:** Robustness Test II.

Variable	(1) *TFP_LP*	(2) *TFP_LP*	(3) *TFP_LP*
*IFDI_mi*	0.0456*** (0.0024)	0.0424*** (0.0021)	0.0339*** (0.0017)
*OFDI_mi*	0.0208*** (0.0036)	0.0177*** (0.0034)	0.0194*** (0.0024)
*IFDI_mi·OFDI_mi*	0.0006 (0.0008)	0.0007 (0.0005)	0.0004 (0.0004)
Controls	NO	NO	YES
Industrial-fixed effect	NO	YES	YES
Year-fixed effects	YES	YES	YES
R^2^	0.1527	0.3293	0.5281
Obs.	24440	24439	24438

Notes: (1) The numbers in parentheses are robust standard errors; (2) *, ** and *** indicate significance at the 10%, 5% and 1% levels, respectively.

**Table 11:** Robustness Test III.

Variable	(1) *TFP_LP*	(2) *TFP_LP*	(3) *TFP_LP*
*IFDI_ma*	−0.2405*** (0.0635)	−0.4022*** (0.0824)	−0.2536*** (0.0673)
*OFDI_ma*	4.5809*** (0.9264)	4.7194*** (1.1878)	3.7417*** (0.9671)
*IFDI_ma·OFDI_ma*	2.6289 (4.1667)	−8.4550 (5.3640)	−10.6360** (4.3671)
Controls	NO	NO	YES
Industrial-fixed effect	NO	YES	YES
Year-fixed effects	YES	YES	YES
R^2^	0.0186	0.0449	0.3671
Obs.	24439	24438	24437

Notes: (1) The numbers in parentheses are robust standard errors; (2) *, ** and *** indicate significance at the 10%, 5% and 1% levels, respectively.

First, the sample period is rescheduled. Given that the sample period used in the benchmark regression model spans from 2011 to 2021, to mitigate the potential impact of the COVID-19 pandemic in 2020 and thereafter on the regression results, data from 2011 to 2019 are used for re-regression, as shown in Models 1–3 of Table 9. Whether it is IFDI, OFDI, or their interaction term, all have a positive effect on the improvement of firms’ total factor productivity and are significantly positive at the 1% level. This indicates that, excluding the impact of the pandemic, whether it is unidirectional capital flow or bidirectional collaborative effects, FDI has a positive promoting effect on total factor productivity under the dual efficient flow of advanced technology and high-value capital.

Second, the dependent variable is replaced. The total factor productivity (*TFP_OP*) measured by the OP method is used to replace the dependent variable (*TFP_LP*) in the benchmark model, which basically maintains results consistent with the benchmark regression model, further verifying the robustness of the model.

Third, to further clarify the robustness of benchmark model, this paper adds the clustered standard errors into the original model. The results are displayed in Table 10. Model (1) to Model (3) include the year-fixed effects, industrial-fixed effect and firm-level controls, accordingly. It is clear that the inward FDI and outward FDI truly stimulate the ascending process of firms’ TFP, which is also consistent with the benchmark model.

Finally, information acquisition and other issues, have a smaller impact of core variables on their production and operation efficiency compared to the macro-regional level and need to be further improved. The annual ratio of foreign direct investment or outward direct investment to regional gross product in the area where the listed firm is located is used as a macro-level measure to conduct robustness checks on the relationship between two-way FDI and firms’ total factor productivity, as shown in Table 11. The empirical results show that the level of regional outward direct investment and foreign direct investment, as well as their interaction term, have a more significant promoting effect on firms’ total factor productivity, consistent with the conclusions of the benchmark model, but exceeding the coefficients of the benchmark regression. This situation indicates that the regional economic agglomeration effect makes the level of international capital flow have a more obvious incentive and promoting effect on the regional comprehensive economic development level. In opposition, firms, limited by their financing and investment structure, organizational structure, information acquisition and other issues, have a smaller impact of core variables on their production and operation efficiency compared to the macro-regional level and need to be further improved.

### 6.4. Heterogeneity analysis

In this section, this paper will conduct heterogeneity tests from the aspects of the region where the listed companies are registered, the specific industry in which the companies are located and the nature of the companies themselves.

#### 6.4.1. Regional heterogeneity analysis.

Given the inherent differences in factor endowments among Chinese provinces, there are significant disparities in the levels of scientific and technological innovation and economic development across regions. These discrepancies may lead to variations in the impact of two-way FDI on firms’ total factor productivity. Therefore, this study divides the full sample into three major regions based on the registered locations of listed firms: the eastern region (including Beijing, Hebei, Tianjin, Liaoning, Jiangsu, Zhejiang, Shanghai, Fujian, Shandong, Hainan and Guangdong), the central region (including Jilin, Heilongjiang, Anhui, Jiangxi, Shanxi, Henan, Hubei and Hunan) and the western region (including Sichuan, Chongqing, Guangxi, Guizhou, Yunnan, Shaanxi, Gansu, Ningxia, Inner Mongolia, Qinghai, Xinjiang and Tibet). Separate regressions are conducted for each region and the results are shown in Table 12.

**Table 12:** Heterogeneity test I.

Variable	Region heterogeneity								
	East			Middle			West		
	(1)	(2)	(3)	(4)	(5)	(6)	(7)	(8)	(9)
*IFDI_mi*	0.0445*** (0.0014)	0.0407*** (0.0012)	0.0328*** (0.0010)	0.0516*** (0.0028)	0.0429*** (0.0025)	0.0298*** (0.0021)	0.0445*** (0.0033)	0.0424*** (0.0030)	0.0345*** (0.0026)
*OFDI_mi*	0.0211*** (0.0016)	0.0159*** (0.0014)	0.0176*** (0.0012)	0.0136*** (0.0033)	0.0132*** (0.0029)	0.0162*** (0.0024)	0.0295*** (0.0037)	0.0249*** (0.0033)	0.0271*** (0.0029)
*IFDI_mi·OFDI_mi*	0.0007** (0.0004)	0.0008** (0.0003)	0.0004 (0.0003)	−0.0006 (0.0008)	−0.0000 (0.0007)	0.0005 (0.0006)	0.0008 (0.0010)	0.0015* (0.0009)	0.0005 (0.0008)
Controls	NO	NO	YES	NO	NO	YES	NO	NO	YES
Industrial-fixed effect	NO	YES	YES	NO	YES	YES	NO	YES	YES
Year-fixed effects	YES	YES	YES	YES	YES	YES	YES	YES	YES
R^2^	0.1528	0.3438	0.5509	0.1513	0.3949	0.5788	0.1486	0.3495	0.5096
Obs.	17257	17256	17255	3957	3956	3956	3226	3224	3224

Notes: (1) The numbers in parentheses are robust standard errors; (2) *, ** and *** indicate significance at the 10%, 5% and 1% levels, respectively.

By comparing the regression results of the three regional sub-samples, it can be observed that IFDI has a positive effect on TFP across all regions. However, the magnitude of this effect is smaller in the eastern and central region compared to the western regions. This may be attributed to the frequent capital flows and intense competition among firms in the eastern or central region, which may limit the productivity-enhancing effects of IFDI. Nevertheless, the slower pace of capital flows in the central and western regions allows for a more pronounced positive impact of IFDI on productivity.

When it comes to corporate OFDI, there are distinct trends across different regions. In this study, corporate OFDI investments tend to also have positive effect on the production and operation levels of firms among three sub-regions, while the influence is most significant in the western region. This phenomenon may be explained by the following factors: In the eastern or central region, when firms allocate their extra production or capital resources to outward investment activities, they tend to form a “quantity-over-quality” expansion, which means that the outward investment may divert resources away from core production activities, resulting in a loss of economic efficiency and proficiency. Therefore, the impact of outward investment on corporate TFP is smaller and limited. On the contrary, in the western region, due to the relatively scarce resources, firms in this area address more on the significance of technological innovations and production elevation, which helps to facilitate the acquisition of reverse technology spillovers, thus promoting production and operation development.

#### 6.4.2. Industry heterogeneity analysis.

Following previous literature [48, 56], which classify industries based on pollution intensity, this study categorizes the industries of listed companies into high-pollution and non-high-pollution industries. To be more specific, 12 industries, including coal mining and selection, non-ferrous metal ore mining, textile industry, paper-making industry, petroleum processing, chemical fiber, chemical fiber manufacturing, non-metallic mineral products manufacturing, non-ferrous metal smelting and pressing, power generation and others, are identified as high-pollution industries, while the remaining industries are classified as non-high-pollution industries. Table 13 accordingly reports the specific regression results for listed companies in high-pollution and non-high-pollution industries.

**Table 13:** Heterogeneity test II.

Variable	Industry heterogeneity					
	High pollution			Non-high pollution		
	(1)	(2)	(3)	(4)	(5)	(6)
*IFDI_mi*	0.0494*** (0.0023)	0.0080*** (0.0012)	0.0325*** (0.0019)	0.0441*** (0.0013)	0.0424*** (0.0012)	0.0337*** (0.0010)
*OFDI_mi*	0.0238*** (0.0027)	0.0071*** (0.0015)	0.0252*** (0.0022)	0.0203*** (0.0015)	0.0164*** (0.0013)	0.0178*** (0.0011)
*IFDI_mi·OFDI_mi*	0.0012* (0.0006)	−0.0003 (0.0003)	0.0011** (0.0005)	0.0006 (0.0004)	0.0005* (0.0003)	0.0003 (0.0003)
Controls	NO	NO	YES	NO	NO	YES
Industrial-fixed effect	NO	YES	YES	NO	YES	YES
Year-fixed effects	YES	YES	YES	YES	YES	YES
R^2^	0.2021	0.8790	0.5039	0.1440	0.3288	0.5434
Obs.	4972	4908	4969	19468	19466	19465

Notes: (1) The numbers in parentheses are robust standard errors; (2) *, ** and *** indicate significance at the 10%, 5% and 1% levels, respectively.

The results demonstrate that the positive effect of IFDI on production efficiency is more pronounced in companies within non-high pollution industries. IFDI in these less polluting firms brings advanced production technologies, R&D capabilities and sophisticated management techniques. Furthermore, these domestic firms in non-high pollution sectors are better positioned to learn from and imitate these cleaner technologies, leading to significant productivity gains.

However, the productivity effect of OFDI exhibits a slightly higher increase in those high-pollution industries. As an economics develops, its environmental regulations typically become much stricter. For high-pollution firms, complying with these regulations at home is extremely costly. By investing overseas such as in countries with less stringent environmental standards, they could avoid these high compliance costs. This immediate reduction in operational costs directly and significantly boosts their measured productivity and profitability. The interactive effect of two-way FDI on TFP is positive for both types of companies, consistent with the benchmark model.

#### 6.4.3. Firm heterogeneity analysis.

In addition to regional and industry differences, firm heterogeneity is another crucial factor to consider in the context of two-way FDI flows. Therefore, this study further distinguishes the full sample of firms into state-owned enterprises (SOEs) and non-state-owned enterprises (non-SOEs) and conducts separate model regressions. The results are presented in Table 14.

**Table 14:** Heterogeneity test III.

Variable	Firm nature					
	SOE			Non-SOE		
	(1)	(2)	(3)	(4)	(5)	(6)
*IFDI_mi*	0.0540*** (0.0022)	0.0513*** (0.0019)	0.0394*** (0.0016)	0.0353*** (0.0013)	0.0339*** (0.0012)	0.0279*** (0.0010)
*OFDI_mi*	0.0363*** (0.0026)	0.0326*** (0.0023)	0.0314*** (0.0019)	0.0098*** (0.0014)	0.0103*** (0.0013)	0.0129*** (0.0011)
*IFDI_mi·OFDI_mi*	−0.0012** (0.0006)	−0.0012*** (0.0005)	−0.0011*** (0.0004)	0.0008** (0.0004)	0.0009*** (0.0003)	0.0007** (0.0003)
Controls	NO	NO	YES	NO	NO	YES
Industrial-fixed effect	NO	YES	YES	NO	YES	YES
Year-fixed effects	YES	YES	YES	YES	YES	YES
R^2^	0.1887	0.4249	0.5926	0.1250	0.3110	0.5039
Obs.	7758	7757	7757	15591	15591	15590

Notes: (1) The numbers in parentheses are robust standard errors; (2) *, ** and *** indicate significance at the 10%, 5% and 1% levels, respectively.

Models 1 and 4 present that the level of IFDI has a positive productivity effect on all types of firms, which is significant at the 1% level. This indicates that foreign investment generally enhances the productivity of firms regardless of their ownership structure. Model 2 reveals that, without controlling for firm size, the outward investment level of SOEs has a positive productivity effect. However, this effect becomes insignificant when firm size is controlled for. This suggests that the size of SOEs, which partly determines their ability to control capital flows and attract government resources, plays a significant role in their outward investment activities. In opposite, small and medium-sized firms, compared to large ones, face greater financing and bankruptcy pressures, which perhaps limit the positive impact of international capital flows on their production and operations.

Model 5 indicates that the outward investment amount of non-SOEs has a negative effect on TFP, although this negative effect is smaller in magnitude than the positive effect of outward investment by SOEs. When firm size is not controlled, the productivity effect of outward investment by non-SOEs is no longer significant. This implies that larger firms, due to their scale economies, can offset some of the negative productivity impacts of outward investment. Once firm size is controlled, this scale effect no longer effectively influences the group of non-SOE listed firms. Models 3 and 6 illustrate that the interaction term of two-way FDI has a significant positive productivity effect, which is more pronounced for SOEs. This may be related to their better access to policy information and broader financing channels. In summary, the analysis highlights the importance of considering firm heterogeneity in understanding the impact of two-way FDI on productivity. The effects of FDI vary depending on whether the firm is state-owned or not and the size of the firm also plays a significant role in mediating these effects.

### 6.5. Moderating analysis

This section presents the moderating effects of firms’ environmental information disclosure quality and technological innovation on the relationship between two-way FDI and total factor productivity. In Table 15, Model 1 indicates that while IFDI positively promotes TFP, the quality of environmental information disclosure significantly enhances this positive effect at the 1% level. This suggests that IFDI directly affects the economic efficiency of individual listed firms by introducing advanced technologies, management practices and talent, thus fostering healthy innovation and market competition among domestic firms.

**Table 15:** Moderating effect of EDM.

Variable	(1) *TFP_LP*	(2) *TFP_LP*	(3) *TFP_LP*
*IFDI_mi*	0.00324^***^ (0.000752)		
*OFDI_mi*		−0.00534^***^ (0.000952)	
*IFDI_mi·OFDI_mi*			0.00293^***^ (0.000325)
EDM*IFDI_mi	0.000285^***^ (0.000065)		
EDM*OFDI_mi		0.000544^***^ (0.0000882)	
EDM*IFDI_mi·OFDI_mi			0.000434^***^ (0.0000278)
Controls	YES	YES	YES
Industrial-fixed effect	YES	YES	YES
Year-fixed effects	YES	YES	YES
R^2^	0.7807	0.7794	0.4914
Obs.	28,145	28,145	28,145

Notes: (1) The numbers in parentheses are robust standard errors; (2) *, ** and *** indicate significance at the 10%, 5% and 1% levels, respectively.

Notably, compared to the results in the benchmark regression, Model 2 shows that the effect of OFDI on production efficiency is weakened under the moderating effect of environmental information disclosure quality. This implies that the quality of environmental information disclosed by firms significantly influences the direction of the relationship between OFDI and TFP. Specifically, environmental constraints faced by firms can significantly undermine their confidence in outward investment and overseas market expansion, leading them to allocate more resources to internal accumulation and development.

Model 3 demonstrates a significant positive moderating effect of environmental information disclosure quality on the relationship between two-way FDI and TFP. This indicates that as listed firms grow and their potential for development increases, the quality of their environmental information disclosure also improves steadily. This, in turn, accelerates the flow of two-way FDI capital, further enhancing the firms’ production efficiency. It is also noteworthy that technological innovation helps to motivate the positive impact of two-way FDI on corporate TFP [38–39]. In Table 16, the coefficients of the interaction of technological innovation and IFDI, OFDI and two-way FDI are all positive and significant at the 1% statistical level.

**Table 16:** Moderating effect of Innovation.

Variable	(1) *TFP_LP*	(2) *TFP_LP*	(3) *TFP_LP*
*IFDI_mi*	0.0040*** (0.0010)		
*OFDI_mi*		−0.0192*** (0.0013)	
*IFDI_mi·OFDI_mi*			0.0016*** (0.0003)
Inno*IFDI_mi	0.0113*** (0.0003)		
Inno*OFDI_mi		0.0181*** (0.0004)	
Inno*IFDI_mi·OFDI_mi			0.0018*** (0.0001)
Controls	YES	YES	YES
Industrial-fixed effect	YES	YES	YES
Year-fixed effects	YES	YES	YES
R^2^	0.5486	0.5156	0.5111
Obs.	24438	24438	24438

Notes: (1) The numbers in parentheses are robust standard errors; (2) *, ** and *** indicate significance at the 10%, 5% and 1% levels, respectively.

## 7. Discussion

China has always been resolute and unwavering in its continuous advancement of high-level opening-up and has been an advocate for the development of an open economy, in order to jointly propel the high-quality growth of the global economy. The existing literature focuses on the impact relationships between any two or three of these variables, which are environmental information disclosure, technological innovation, TFP and FDI [14, 49, 57], yet fails to construct a more precise analytical framework that encompasses all core variables. As a result, the complex interactive mechanisms among these crucial variables remain unclear. Our empirical studies represent that both inward foreign direct investment and outward foreign direct investment exert positive and significant impact on firms’ TFP with statistical significance. It can be clearly attained that the effect of OFDI on TFP is slightly smaller than that of IFDI, suggesting that on average, Chinese listed firms’ inward investments are more essential to elevating their productivity than IFDI does. Furthermore, IFDI can introduce new technologies and management practices that improve production efficiency. Meanwhile, it reflects that firms’ outward investment or financing activities significantly boost their economic efficiency and development, generating a reverse technology spillover effect, but still in an initial stage.

Consistent with Environmental Kuznets Curve (EKC) theory, our empirical results illustrates that when a country is underdeveloped, environmental pollution is relatively mild [12, 18]. However, as per capita income rises, pollution levels increase, worsening environmental degradation. Once the economy reaches a certain threshold or “turning point”, further increases in per capita income lead to a decline in pollution levels and an improvement in environmental quality. The majority of existing studies analyze the impact of FDI on TFP from a macroscopic national or provincial level or a meso-level industry perspective [2, 20, 45]. On the contrary, analyses of key economic variables at the micro-level firm-specific level are relatively scarce.

Up till now, the academic community has not yet established a unified measurement standard for TFP. There is still considerable debate over whether different measurement methods will ultimately affect the robustness of empirical conclusions. Moreover, research on the construction of corporate environmental disclosure quality systems is also underdeveloped and requires further discussion. This paper adopts the environmental disclosure framework from the CSMAR database to comprehensively evaluate firms’ environmental disclosure capacity. While simultaneously being presented with additional development opportunities, environmental disclosure quality and technological innovations in turn exerts complex moderating effects on the improvement of total factor productivity, by integrating two-way FDI, corporate environmental information disclosure and total factor productivity into a unified research framework.

The heterogeneity analysis results elucidates that, compared with the eastern or central region, the productivity-promoting effect of IFDI is higher in the western regions. In the meantime, corporate OFDI investments tend to also have positive effect on the production and operation levels of firms among three sub-regions, while the influence is most significant in the western region. The positive effect of IFDI on production efficiency is more pronounced in companies within non-high pollution industries. However, the productivity effect of OFDI exhibits a slightly higher increase in those high-pollution industries.

As an economy develops, its environmental regulations typically become much stricter. The interactive effect of two-way FDI on TFP is positive for both types of companies, consistent with the benchmark model. This study clarifies the effects and mechanisms of two-way FDI on firms’ TFP. We conclude that environmental disclosure quality and technological innovation could directly positively moderate the influence of two-way FDI on firms’ TFP [57]. Given the stronger productivity-enhancing effect of IFDI in the western region, policy should focus on attracting high-quality inward FDI to these areas. This includes offering targeted tax incentives, infrastructure support, and streamlined administrative procedures to facilitate technology spillovers and management expertise transfer.

## 8. Conclusion

This study systematically elucidates that against the backdrop of the era of “carbon peak” and “carbon neutrality”, how the international capital flow is subject to certain environmental constraints. Based on previous studies that have predominantly focused on the impact of international investment flow on green TFP at the provincial or regional level, this paper further explores the effects of two-way FDI and its interactive relationship on TFP of listed companies from a micro perspective, thus enriching the research perspective on the interplay between environmental protection and economic growth. Take A-share Chinese listed companies from 2011 to 2022 as research samples, this paper discusses the effects and potential moderating mechanisms of two-way FDI on corporate total factor productivity. Firstly, It is empirically found that the impact of corporate IFDI on its own total factor productivity is higher than that brought by OFDI. After a series of robustness tests, the baseline results are still firm and valid. Secondly, moderating analyses demonstrate that both corporate environmental information disclosure quality and technological innovations exert significant moderating effects on the relationship between two-way FDI and corporate total factor productivity. Thirdly, this paper finds that the effects of two-way FDI faced by listed companies in the central and western regions, non-heavily polluting companies and state-owned listed companies are more significant.

Due to the limitations of data collection and sample size, we only incorporate Chinese A-share listed firms as our study targets. Further researches could emphasize more on other kinds of firms such as small and medium-sized firms, in order to get a more comprehensive understanding of the effect of capital flow on TFP. Moreover, information about different destinations of OFDI could be specified in order to track outward capital flows and its socioeconomic implications across nations. Relying on the empirical analysis above, this paper provides multiple relevant policy recommendations to stimulate the impact of the production effect of international capital flows.

First, implement differentiated strategies for two-way FDI utilization. Encourage enterprises to actively introduce high-quality IFDI, particularly in advanced manufacturing and green technology sectors. Support qualified firms in conducting OFDI in technology-developed countries, establishing overseas R&D centers, and facilitating reverse technology spillovers. Provide tailored policy assistance for small or middle sized enterprises, including financial services, investment consulting, and risk mitigation. Establish a monitoring database to track the impact of two-way FDI on total factor productivity.

Second, enhance region-specific policy support to leverage FDI effectiveness in central and western regions. Strengthen policy incentives for attracting IFDI to these areas through tax benefits, land support, and infrastructure development. Establish collaboration channels between eastern and western enterprises to promote joint OFDI initiatives.

Third, adopt industry-specific guidance to achieve green and efficiency gains. Direct IFDI investment toward high-tech, digital and low-carbon sectors in non-highly polluting industries. Promote reverse technology spillovers through OFDI Support. While the impact of OFDI on TFP is currently weaker than IFDI, policy should encourage firms, especially in high-pollution sectors, to invest abroad in technology-intensive industries. Support mechanisms such as overseas investment risk insurance, strategic partnership programs, and post-investment technology integration assistance can amplify reverse spillover effects.

Fourth, develop a comprehensive support system to optimize the investment environment. Improve corporate environmental information disclosure and regulatory compliance. Strengthen environmental disclosure standards and enforcement. Since environmental disclosure quality moderates TFP improvement, regulatory bodies should establish mandatory, standardized corporate environmental disclosure frameworks.
